# Nurses in France: Between Autonomy and Subordination in Front Line Care

**DOI:** 10.1177/2333393615584550

**Published:** 2015-05-14

**Authors:** Ljiljana Jovic, Evelyne Bianchi, Sylvie Decouflet, Valérie Loizeau, Patricia Amiot, Maria Teixeira

**Affiliations:** 1 Univ Paris Diderot, Sorbonne Paris Cité, UMR-S 1123, F-75019 PARIS, France; 2 Agence Régionale de Santé Ile-de-France, Paris, France; 3 Institut de Formation en Soins Infirmiers, Paris, France; 4 Assistance Publique Hopitaux de Paris, Hopital Robert Debré, F-75019 Paris, France; 5 Centre de Santé, Direction de l’Action Sociale, de l’Enfance et de la Santé, Mairie de Paris, France; 6 Centre Hospitalier Intercommunal Poissy-Saint-Germain, France

**Keywords:** anthropology, health care, primary, health care professionals, nursing, sociology

## Abstract

In France, medical practitioners are aware that the practice of the delivery of primary care by nurses occurs in other countries. However, there is disagreement about how to implement this practice. This aspect of the issue of front line care has not yet been studied in France. In this article, our aim is to identify to what extent the delivery of primary care by nurses is considered acceptable by doctors and nurses working in hospital emergency departments and in public and private health centers. The results of our research provide a picture of opinions that exist among doctors and nurses. These opinions highlight practices that are outside the current regulations and present perspectives, which range from conditionally in favor to unfavorable. Such opinions contribute to our knowledge because they are derived from the professionals directly involved and describe what is acceptable in this particular context.

## Introduction

Emergency departments today receive increasing numbers of patients with general medical complaints. This trend is not unique to France but has long been observed internationally (Boyle, Beniuk, Higginson, & Atkinson, 2012; Schneider, Gallery, Schafermeyer, & Zwemer, 2003; Tahan & Cesta, 2005). A poor distribution of health professionals across the country, aging of the population, and the growth of chronic disease are real problems that are overloading emergency departments and limiting access to care. Apart from emergency departments, other health care organizations and groups of health care professionals, such as public health centers and private centers, are seeing types of patients whose need to be seen by a doctor may be debatable.

The organization of care and the enactment of the competences and capacities of health professionals must be reviewed to safeguard the quality of care given to all patients, especially to those who arrive in a life-threatening condition or emergency, those whose condition is likely to deteriorate quickly, or those presenting with complex clinical conditions (Bodenheimer, 2008). In other countries, advanced practice nurses are responsible for delivering front line care. Research shows that first response care given by nurses is as effective as that provided by doctors (Keleher, Parker, Abdulwadud, & Francis, 2009).

The existence elsewhere of forms of advanced nursing practice is known in France and is even cited or recommended in reports (Berland & Gausseron, 2002, Berland, 2003; Cordier, 2013; Hénart, Berland, & Cadet, 2011). However, these types of advanced nursing practices are not yet formalized; thus, forms of advanced nursing practice have yet to be developed in France. There is also said to be resistance to reforming the nursing profession. At present, there are no data to enable us to objectively measure the acceptability of a transformation of the profession that would place nurses on the front line of delivering primary care.

Public health authorities are exploring the advantages and the possibilities of organizing advanced nursing practice and of creating new professional pathways to fill the gaps left by changing health care needs. These issues have been the subject of several ministerial reports; however, concrete proposals are lacking. Until the present, there has been no French definition of advanced nursing practice. Usually, discussion about this topic refers to the work of the International Council of Nurses (2009):

[a] Nurse Practitioner/Advanced Practice Nurse is a registered nurse who has acquired the expert knowledge base, complex decision-making skills and clinical competencies for expanded practice, the characteristics of which are shaped by the context and/or country in which s/he is credentialed to practice. A Masters degree is recommended for entry level. (p. 1)

At the international level, nurses’ responsibilities are not uniform, in spite of this international definition of advanced practice. Thus, each country can adapt the implementation of this definition to its context.

### Aim of the Study

The aim of this article is to identify the acceptability, in the view of doctors and nurses working in emergency departments, public health centers, and private health centers, of advanced care being delivered by nurses. Is the nursing profession ready to accept more autonomy in front line care? Intentionally, we have not used the expression “advanced nursing practice” in our guidelines, focusing the questions on their everyday practices to see whether they have activities outside the nursing regulations.

## Background

In some countries, nurses play an independent role in front line care. They are sometimes called “advanced nurse practitioners” or “specialized clinical nurses.” In the United States, these practices were established and formalized in the first half of the 20th century. Later, other countries, such as Canada, also adopted this pathway (DiCenso et al., 2010; Kaasalainen et al., 2010). In the United Kingdom, the “new nursing” movement has been working for some years toward greater autonomy for nurses (Feroni & Kober, 1995), and advanced practice has become operational (Mc Gee & Castledine, 2003). In France, these professional roles do not exist in the same way, nor is there the advanced nursing practice that has generally developed in other countries, such as the United States (Donelan, DesRoches, Dittus, & Buerhaus, 2013; Rigolosi & Salmond, 2014), where the Nurse Practitioners Modernization Act will take effect on January 1, 2015.

In other countries, with contexts as varied as the United Kingdom, such as the Republic of Ireland, Switzerland, Jordan, Pakistan, Holland, Spain, Sweden, Lebanon, New Zealand, and Australia, work is ongoing to establish and develop advanced nursing practice and to create educational programs for this practice. In French-speaking Switzerland, a consensus about the definition of advanced practices is emerging (Morin, Ramelet, & Shaha, 2013). It is true that the organization of care in each country has an important impact on the evolution of the nursing profession.

In some countries (Belgium, France, Ireland, Czech Republic), the principal mode of delivery of front line care continues to depend mainly on medical doctors alone, while in other countries (Australia, Canada, United States, Finland, UK), clinical practice teams are the main model . . . . As might be expected, advanced practice by nurses in front line care is most developed in the countries where primary care is mainly provided by clinical practice teams. (Delamaire & Lafortune, 2010, p. 55, Authors’ translation)

Therefore, the specificity of each country must also be taken into account.

The French concept of a “particular role” assigned to nurses appears for the first time in a law passed in 1978 (Acker, 1991, p. 135). Essentially, this role consists of providing personal care for the comfort and life support of the patient (Collière, 1982, 1996). These forms of care have been codified and given a theoretical basis: “nursing” as a profession also includes education, surveillance, and support for patients and their families. Within this framework, “the nurse is competent to take initiatives and carry out the care he or she judges to be needed” (Article R.4311-3 of the Code de santé publique—Public Health Code, Authors’ translation). However, this autonomous space confines nurses within a compartment in a limited sector. Outside this particular role, nurses also put doctors’ prescriptions into practice in conformity with the French Public Health Code. A nurse’s activities may take any of three general forms: (a) a prescription involving more or less close supervision by a doctor, ranging from the physical absence to the presence of the doctor in the immediate vicinity at the time the action is performed; (b) formalized prescriptions in the form of a protocol giving the nurse the freedom to determine the application of a course of action, according to predetermined criteria; and (c) the retroactive authorization of actions carried out in an emergency and in the absence of a doctor. In the French system, care of whatever type is always delivered on medical authority, with rare exceptions. However, the distinction between actions performed as part of the nurse’s “particular” role and those that follow a doctor’s prescription is a formal one. The practice is more complicated. Some practices, which partly correspond to the characteristics of advanced practice, have been observed (Jovic, Guenot, Naberes, & Maison, 2009). In France, the debate on these subjects truly began at the national level in the early 2000s. France has been slow in establishing advanced practice. From the French point of view, it is considered daring for a nurse to take care of a patient without the supervision of a doctor. However, changes in health needs, professional practice, and the economics of health care are raising questions about the sustainability of this model and about its possible reform. There are child care, operating theater, or anesthesia nurses, with specific pay levels, but their status in relation to medical procedures is no different from that of generalist state-registered nurses.

## Method

### Design

To achieve our research aim, we used a qualitative methodology. This type of approach is more suitable for exploring a theme that is poorly known. With no a priori assumption of what we will find, we adopted a perspective of open description. After data collection, in line with the themes that emerged, we decided not to analyze whether the change was possible but whether it was acceptable.

### Research Sites and Participants

The qualitative field research took place in 2011 and 2012 in Paris and the Paris region in seven emergency departments that dealt with patients with medical (three), psychiatric (two), and/or pediatric (two) problems; and in three public health centers and two private health centers. In the first two of these structures, the health professionals are salaried public servants, whereas in the third, they are independent and work in the private sector. The objective of this sampling was to present a diversity of health institutions representative of the health organization of the region in rural and urban areas.

The interviewed sample (*N* = 24) consisted of nurses (*n* = 12) and doctors (*n* = 12). The specialties of the nurses and doctors were as follows: medicine (*n* = 8), psychiatry (*n* = 2), and pediatrics (*n* = 2; see Table 1).

**Table 1.** Demographic Characteristics of the Participants.

Characteristics	Nurses	Doctors
Female	12	5
Male	0	7
Average age	43 (range = 32–55)	43 (range = 34–54)
Length of service in years		
<2	1	0
2–5	4	4
6–10	6	3
11–15	0	4
16–20	0	0
>20	1	1

To be included in the study, the health workers were required to have been in the service for at least 2 years; despite this, one of the nurses had been on the team for less than 2 years, but was nevertheless its longest serving staff member.

### Data Collection

Data were collected via in-depth interviews (Blanchet & Gotman, 1992; Pires, 1997), which are suitable for a socio-anthropological type of analysis. For a phenomenological analysis, approximately 10 interviews are sufficient (Savoie-Zajc, 2007), but for data saturation, generally, at least 20 are needed. “Saturation means that no additional data are being found whereby the sociologist can develop properties of the category. As he sees similar instances over and over again, the researcher becomes empirically confident that a category is saturated” (Glaser & Strauss, 1973, p. 61). After 24 interviews, we reached this point. This qualitative methodology is based on an inductive approach, identifying themes that emerge spontaneously from respondents’ examples of activity.

Two semi-structured guides were developed, one for the doctors’ and one for the nurses’ interviews (see appendix). These guides were similar, but were adapted to each respondent category to collect the different perspectives of professionals regarding the acceptability of nurses being more autonomous and dealing with patients independently.

The interviews, which were between 30 minutes and 1 hour 30 minutes in duration, were carried out face to face (Olivier de Sardan, 2003). They aimed to capture opinions regarding greater autonomy for nurses in their professional activities, particularly in the provision of first response care.

### Data Analysis

The interdisciplinary approach, combining nursing science and anthropology, made it easier to adopt a neutral point of view when confronting different perspectives in the analysis process. The analysis of the verbatim transcripts was structured using Nvivo10 software. Repeated readings of the respondents’ statements enabled emergent themes to be identified. Subsequently, units of meaning were created that could be hierarchized in a problem tree analysis. The themes pertained to different units of meaning: the notion of accepting a wider competence in front line care under the supervision of a physician or without it, knowledge that nurses have or lack, and their different activities. This tree was discussed among the research team. Recurrent themes, and their variations from one account to another, were analyzed according to the characteristics of the interviewees and their differing socio-demographic and thematic attributes. From the thematic point of view, it was determined whether the interviewee was generally practicing outside the regulations, or had opinions conditionally in favor, or unfavorable toward the idea of nurses taking sole charge of patients in situations that, in their opinion, do not require the attention of a doctor. The socio-demographic attributes were sex, age, length of service, medical specialty, and type of health care organization. The data collected could be cross-checked, associated, grouped, discriminated, and related with reference to these different attributes. In this way, it was possible to compare different segments of discourse and to analyze them using a socio-anthropological method that considers the particularities and the lived experience of the speakers (Paillé & Mucchielli, 2005). Each quotation cited here shows the socio-demographic characteristics of the speaker in brackets at the individual’s first appearance in the text.

### Rigor

The guidelines were developed by a group of seven nursing researchers in the ARS (Agence Régionale de Santé) of the Ile-de-France region. They were tested in a pilot study involving five nurses and four doctors. This pilot study enabled the team to readjust the original guideline. After validation of the interview schedules, the interviews were conducted by an anthropologist. The identity of the fieldwork researcher made the professionals feel more comfortable because the anthropologist was independent and outside the institution. The research findings and the analyses were discussed collectively by the members of the research team.

### Ethical Considerations

In France, ethical approval is not required for this type of research. We obtained the authorization of the direction of each institution. In each one, respondents were informed of the research objectives by their managers. On the day of the meeting, the anthropologist again explained the objective of the research, to gather their opinion on a change in the role of nurses, and obtained consent from each interviewee at his or her place of work. All interviewees were volunteers, could withdraw from the interview, and were not obliged to respond to all questions. Interviews were recorded, anonymized using fictitious names, and transcribed in full. The recorded data and the transcripts are stored in an electronic database and will be destroyed 5 years after the end of the research. Quotations from interviews in this article have been translated into English by the author.

## Results

Respondents did not refer spontaneously to advanced practice competencies because this category is not well known by the nurses and physicians themselves. Some of the interviews reflect points of view unfavorable toward the suggested change in the nursing profession; others accept practices outside the regulations and are in favor of this type of development; and some are highly ambivalent, developing opinions that are conditionally in favor. However, regardless of the opinion of the interviewee, what emerges is an identification of the conditions that would have to be fulfilled before such a development could take place. Half of the doctors were accepting toward practices outside the regulations, and so were more than half of the nurses. It is interesting to note that the responses of male doctors accounted for all unfavorable reactions among the doctors. Of course, our qualitative methodology means that we cannot interpret this finding to generalize this difference between male and female perspectives.

### Practices Outside the Regulations

In actual practice, nurses go beyond their official responsibilities in all sectors of activity. They take initiatives that they regularize after the event, where necessary, by requesting retroactive medical prescriptions for them. In doing this, some nurses are interpreting and adapting rigid protocols that do not always align with the realities of their workplace, or with the particular circumstances of a patient. Others go further and take sole charge of patients who later leave the hospital without having seen a doctor. In France, each patient arriving in an emergency treatment center must be seen by a physician as a matter of principle. However, Beatrice sometimes receives patients whose health condition is not serious, according to her. She attends to them, then lets them go, informs the doctor after the event, and then writes the episode up in the patient file. Talking about a person with no fixed address, who is well known and monitored by the health service, she said,

Madame T, when she comes in . . . we almost greet each other with a kiss on the cheek. So I say to her “So, what is the problem? Would you like to eat something?” “Yes, please,” and that’s it. We have a little chat, I give her something to eat and off she goes. And so she doesn’t see the psychiatrist. (Beatrice, aged 44, psychiatric emergency nurse)

Some doctors highlighted nurses’ competence in evaluating the clinical condition of patients. “When you have a patient in the middle of a bronchiolitis episode, for example, the hospital nurses have enough know-how to evaluate the seriousness of the child’s condition at a glance” (Oscar, aged 44, general practitioner in a public health center).

In a health center, patients are normally seen by appointment. However, people do come in without appointments. The nurses give advice to those who present with health conditions that they judge not to be serious. Sometimes, by giving advice, they manage to reassure these patients to the point where they decide to go home and follow the nurse’s advice without seeing a doctor. Nurses working in health centers receive their patients by appointment in the morning or evening for treatment; during the day, they make their home treatment visits. They alert a doctor if they encounter a problem but also go further in anticipating medical prescriptions.

A patient I go to see for other reasons . . . she sprained her arm, so I go to do her bandage and I sort out the prescription later with the doctor in charge. . . . If I have a catheter which is blocked or if I am not sure, if there’s blood, I take it off and remove the catheter and replace it, without prescription, I authorize it later, that can happen. (Charlotte, aged 39, private health center nurse)

Nurses and doctors observe situations where nurses are, in effect, delivering “front line care” in the sense that they decide, on the basis of their analysis of the situation, whether to direct the patient to see a doctor or to administer front line care themselves.

### Opinions Conditionally in Favor

Some of those interviewed were prepared to accept that some primary patient care should be the responsibility of nurses, provided that they receive specific forms of training and that the new practices are closely circumscribed and officially recognized. These changes would have the advantage of providing types of care more appropriate for the needs of the patients, of enabling better use to be made of professional skills, and of having a positive impact in health-economic terms. However, they also highlighted the obstacles, the drawbacks, and the risks involved in making changes in the modalities of cooperation between professionals. Further training could take place after the initial diploma, be tailored to the needs of each service, and depend on the target population. For example, applying plaster casts is a repetitive procedure that, according to Roberto, “doesn’t require knowledge of the whole of human anatomy.” The same goes for dealing with gastroenteritis: “She [the nurse] doesn’t need to know everything about physiopathology, all the human intestinal diseases, it’s enough for her to be able to recognize the signs when it is serious enough to need a doctor’s attention” (Roberto, aged 39, pediatrician in emergency pediatric department).

Certain domains could be prioritized. Health education and dialogue could be offered by the nurse while providing nursing care. In the long term, there would be greater effectiveness and efficiency in the health care system, and time invested by a nurse would have a longer term impact.

It’s a more efficient use of resources, I think, to have a nurse who spends three-quarters of an hour with the parents to do basic care for the child if the child has a fever, rather than for them to see the doctor quickly for 10 minutes and for him to say “What are you doing here? It’s not worth coming to the emergency department for this, it’s nothing,” so that the parents get upset, the doctor gets annoyed, the doctor gets tired out, the parents don’t understand, and then they go to a different hospital, to another emergency department and it just goes on like that. (Roberto, aged 39, pediatrician in emergency pediatric department)

The demand for professional recognition has been a constantly recurring one. Odile (aged 48, nurse in a public health center) regretted that her educational work was invisible. If there was a recognized form of nurse consultation, this would make financial and professional recognition possible, and the work that nurses already do in health centers would become visible.

Micheline (aged 47, general practitioner in a public health center) was in favor of nurses monitoring some chronic diseases. They could certainly adapt and vary the dosage of medicines. They would also be capable of managing adults’ and children’s vaccination records, and in particular, they are unanimously recognized for their skill in bandaging and dressings. Doctors prescribe the medical products needed for dressings as dictated by nurses, and all agreed that nurses would be completely competent to issue these prescriptions themselves. The involvement of nurses in a more autonomous and enlarged role has the potential to improve patient care.

I would like to be able to do certain things. For example, take the case of an asthma attack: I can evaluate, I know he is having an asthma attack, I can give him Ventoline® (a bronchodilator) straight away without waiting for the doctor to tell me “yes, go ahead, you can give him Ventoline®,” that would save everyone’s time. I think the patient’s care would be better. (Odile, aged 48, nurse in a public health center)

Psychiatric nurses could be solely responsible for seeing some patients, for example, those who need an address for a medico-psychological center, who need to talk, or who have a social problem. These professionals, at least the most experienced among them—in other words, those who have knowledge of mental illness and several years of experience in psychiatry—are quite capable of calming such patients and of responding to their anxiety, according to André (aged 28, psychiatrist in psychiatric emergency department of a hospital).

### Unfavorable Points of View

Obstacles and drawbacks of different types and even questions regarding the feasibility of greater autonomy for this class of professionals, as well as resistance to greater delegation of activities to them, were also raised and expressed by both doctors and nurses.

The nurse could . . . do the first reception of some types of demand for direct primary care, but of course, we know that we will wrap ourselves in the flag and say “be careful, it’s risky!” and all that, but no, we really know quite well that there are things which could be taken on by a nurse. (Gérard, aged 53, general practitioner in a private health center)

Some nurses argued that they would require the time to fulfill their current duties first before they expanded their areas of competence. If other responsibilities are given to nurses, there is a risk of them “making mistakes” (Louise, aged 32, medical emergency nurse), although, at the same time, there was recognition that nurses could take more initiative.

According to Anita (aged 40, psychiatric emergency nurse), there would need to be more nurses. She would be prepared to take sole charge of patients, but only on this condition. There is also an issue of professional identity.

I am not a doctor or a frustrated nurse. In the end, I chose to be a nurse; I never went to medical school. I don’t regret not having done this wonderful thing they call inserting stitches, I couldn’t care less. I am a nurse, I give other sorts of care. I have different skills. (Brigitte, aged 35, medical emergency nurse)

The currently available human resources would not allow nurses to give enough time to their other activities. “Minor ailments,” which nurses are supposed to take care of, are not what delays the work, according to Laurent. The option of greater responsibility would not speed up the flow of patients.

To be honest, they [the nurses] already have a lot of work to do. Often, we are waiting for them to be free rather than the other way around, so if they are given even more tasks, I am not sure that will speed up the flow, it will just put them in greater difficulty. (Laurent, aged 47, emergency specialist)

Bruno (aged 40, emergency specialist) argued strongly against a medical practice that would no longer be “two-speed” but “multi-speed medicine,” because different patients would be seen by different professionals who would not have the same level of training and the same skills. In the same way, Patricia wondered about the sense and the appropriateness of such a change in the nurse’s mission in the French context.

In Africa, the competence of a nurse is more or less that of a little doctor, you know? Of course, in situations of emergency or medical vulnerability, paramedics can be trained who can work themselves into a position of diagnostic autonomy. This could be an option. But then I ask myself: “why here?” (Patricia, aged 50, pediatric emergency nurse)

The idea of expanding nurses’ roles in current conditions was, thus, related to the situation in developing countries. Experience as the sole attribute would not suffice. For the status of nurses to change, according to Bruno, there would have to be a solid basis in training because experience can consist only of having done repetitive tasks for a long time and of “bad habits which are rooted in time. Experience doesn’t mean that much.”

For some of the activities already referred to (Roberto, aged 39, pediatrician in emergency pediatric department), an additional training for nurses would not need “to be heavy or long.” However, Bruno (see aged 40, emergency specialist) considered it indispensable to create a training course with access by competition, and with the award of a diploma for those nurses capable of seeing patients on their own, just like the existing French diploma for anesthesia and operating theater nurses. In the current state of nursing training, if a patient is not being seen by a doctor, this is considered a lost opportunity for the sick person. There was plenty of testimony to the effect that important problems might be concealed behind a pathology that seems not to be serious.

The authorities believe that patients come to emergency departments without good reason. One day, they will have to understand that people have reasons for coming here. But because they say it’s for nothing, that’s why they say nurses can just see them quickly. (Bruno, aged 40, emergency specialist)

Another risk is that there will be an inequitable distribution of activities between professionals. Odile (aged 48, nurse in a public health center), who is very involved in setting up protocols to broaden the competence and autonomy of nurses in health centers, expressed her fear and that of her longest serving colleagues that doctors might unload the most unrewarding activities onto them, as has happened in the past.

Today, evacuation of impacted feces is an action which is recognized as one of our AMIs (acte médical infirmier—nurse medical procedure). Well, when I started my career, it was not allowed. OK? It was the junior doctors who had to come round every morning to do that sort of thing.

Brigitte (aged 35, medical emergency nurse) pointed to her own skills and areas of competence and to the specificity of her training.

I have the diagnosis to hand, and I deduce from it what to watch out for. He [the doctor] has the clinical symptoms to hand, and he can deduce the cause. There you have it, right there, at the beginning of year zero of our studies, we are on two completely different tracks. So we each have our own outlook and approach, which is completely different from the other. That’s why I think it’s good for each one to stay in his or her own area of competence.

Pauline (aged 40, general practitioner in a public health center) was concerned about whether nurses with expanded areas of competence would be covered from the medico-legal point of view. In her view, if the status of nurses changed, and if they had the same role as the doctor, the valuation and remuneration of their activities would also have to change; otherwise, the measure would be unjust.

Gaby (aged 58, nurse in private health center) spoke about establishing nurse consultations and, caught between enthusiasm and reservation, exclaimed “Oh yes, personally I would love that.” However, she also supposed that the aim of the State is to save money and went on to complain about her current working conditions: “We have the right to prescribe, for dressings or for blood glucose test strips, but we aren’t paid for prescribing—I resisted doing prescriptions for 3 years. I said ‘I am prescribing but not being paid, that’s not right.’”

Gérard (aged 53, general practitioner in a private health center) also had economic arguments against the idea: If hospitals address the most urgent cases and nurses deal with the least serious, what will be left for the general practitioners?

New forms of collaboration would have an impact on the relationships between doctors and nurses but also on those between paramedical professionals. A pyramid-shaped structure works against the recognition of the competencies that nurses could develop. According to Laurent (aged 47, emergency specialist), nurses sending patients for radiology would come up against resistance from the electro-radiology technicians, who would not accept this. Here, a hierarchical order is also being expressed. If general practitioners sending patients to a psychiatrist for a psychiatric evaluation know that this evaluation will be done by a nurse, this will cause problems.

The general practitioners send us people for an evaluation of suicide risk. If we tell them the evaluation is going to be done by a nurse, there will be a lot of trouble in the system. I mean, even doctors are not sure, even doctors can miss something, after thirty or forty years of experience. (Benjamin, aged 54, psychiatrist in psychiatric emergency department)

Finally, there must not be any conflict between professionals, and nurses have to feel supported by the doctors they work with, which is not always the case. Beatrice (aged 44, psychiatric emergency nurse) described her daily experience in psychiatric emergency:

When there is a problem with a patient, and it boils over and you have to restrain him, well, there are psychiatrists who make themselves scarce. They leave you to it, to take the punch in your face. That’s how it is. So if there were psychs, super-competent and who you could work with, I think I wouldn’t do it any more [seeing patients alone]. At the moment, I feel I am not specially covered by the psychiatrists—they send you to the slaughter, so there’s no reason why I should do their job.

What is going on behind these statements and these shared positions? In the course of the discussion, we will describe the French context.

## Discussion

The findings of the survey allow us to illustrate a number of ideas that are at work when the possibility of the role of nurses moving toward greater autonomy is suggested. An almost identical number of reservations and endorsements of the idea of such a transformation of the profession was expressed, both by the doctors and by the nurses. There was no substantial preponderance in the direction of greater autonomy for nurses or for the status quo in their roles and competencies. When health professionals were asked for their opinions regarding the possibility of nurses receiving and treating patients without those patients being seen by a doctor if the nurses considered this unnecessary, these opinions were highly nuanced, with each respondent leaving the room for further discussion and controversy. The research results indicate the extent to which nurses in France act autonomously or even carry out some activities independently and then seek retroactive authorization from a doctor.

We see in the results that there are practices that go beyond the regulations, activities that are already done, and other activities that could be performed. Some of these activities could constitute “advanced practices.” So after reading the interviews, it seems interesting to analyze these judgments using the concept of acceptability.

Acceptability is a complex concept that does not yet have an agreed on definition. It is used in the work of a variety of domains (new technology, psychology, economics, ecology, and so forth).

Social acceptability . . . results from a judgmental process by which individuals (1) compare the perceived reality with its known alternatives; and (2) decide whether the “real” condition is superior, or sufficiently similar, to the most favorable alternative condition. If the existing condition is not judged to be sufficient, the individual will initiate behavior—often, but not always, within a constituency group—that is believed likely to shift conditions toward a more favorable alternative. (Brunson, 1996, p. 9)

This author further develops this notion of acceptability. From his point of view, the basic criteria used in the evaluation of a comparison of alternatives are desirability, equity, and feasibility. To this individual dimension is added a social dimension, which includes a level of tolerance and environmental and practical conditions. Here, the question is whether a practice respects the internalized norms of a group, and whether the group is prepared to take a risk and to accept its potential consequences.

In reality, the distinction between what is acceptable and what is unacceptable is not clearly drawn. There are zones of nuance. Acceptability may be defined as a form of evaluation based on references that are particular to each person, such as experiences, supposed benefits, values, and individual preferences. The social dimension adds to this individual perspective by introducing interactions between individuals, as well as between individuals and institutions (Stankey & Shindler, 2006; Wüstenhagen, Wolsink, & Bürer, 2007). This judgment is a dynamic and provisional process. It is built up through anticipating the impact that the change might have. Stankey and Shindler (2006) list five categories of factors that influence the judgment of acceptability: (a) the context, which can be spatial, temporal, and/or social; (b) the risks and uncertainties linked to the practice; (c) the aesthetic aspect of the outcome; (d) the trust in decision makers and institutions; and (e) personal knowledge and techniques. These categories were adapted for the purposes of our research. They gave us suggestions for interpreting the opinions of doctors and nurses regarding a new organization of front line care.

### Context

Factors relating to spatial, temporal, and social context reveal that in each type of health structure, there is a form of proximity and collaboration between doctors and nurses. However, this scenario adapts in response to personal initiatives and to individual analyses of the context, leading to practices that go beyond the regulations.

The highly hierarchical context of the hospital works against making nurses autonomous. The hospital was the only structural setting where starkly unfavorable opinions were expressed. These resistances were due more to an ideological position, or to a defense of territory and power. In a gendered perspective, the fact that most recruits to the nursing profession are female encourages certain stereotypes. Women are assumed to be creatures of devotion, who give unstintingly to take care of others and would even do so for nothing.

According to Anne Véga (1997), the hospital is

a place of intrication of collective representations, which are expressed in rituals assigning places, gestures and speech to each person in an extremely precise manner, according to rhythms which are also highly codified. The confrontations of differing logics of power and of prophylactic practices form part of the working environment as well as of bodies. (Véga, 1997, p. 126, Authors’ translation)

For example, in France, nurses lack the prerogative to perform some interventions that are commonly done by nurses in other countries, such as sutures or plaster casts.

Among the obstacles identified were a shortage of time and an insufficient number of staff. Greater autonomy for nurses was interpreted as implying extra work, which could lead to more mistakes in treatment and care. Potential new functions were seen as being additional to activities already being undertaken as part of the nursing role. They were rarely seen as singular and to be performed, at least in part, in the place of current activities, or as enabling some existing practices to become officially recognized.

Nurses and doctors do not oppose the revaluation of nursing. They believe that nurses are capable of widening their domain of competence if they are given the means to do so, and provided with reasons to be motivated and to discover a personal interest that goes beyond the well-being of the patient. However, nurses do not want the most unrewarding activities, which no one wants to do, to be delegated to them, which would only deepen their sense of subordination within a strongly hierarchical system.

Some nurses would be more motivated to acquire new competencies if these were recognized and valued by a change in their status or remuneration, or in the valuation of their activities. For the moment, they engage in training out of personal interest or for the good of their patients, but this extra knowledge is not officially valued. Better recognition of new areas of knowledge they might acquire would be a way of motivating them, particularly by holding out the possibility of progression in their professional careers. Here, too, the use of the skills acquired through training and practice remains a matter of personal initiative.

In terms of context then, acceptability has to do with the competencies to be recognized and valued for nurses to perform activities autonomously in specific and relatively restricted domains. The limitations are mainly linked to the hierarchical and compartmentalized organization of the health professions, especially in hospitals, and to the lack of human resources.

### Risks and Uncertainties

The risks and uncertainties linked to practice were expressed and considered. Several interviewees questioned the utility and interest of a proposal to allow nurses to perform front line care, with some considering this option to be dangerous for patients. Some doctors and nurses believe that all pathologies must be diagnosed by doctors because a problem that appears benign might conceal a serious medical condition. The proposed change would mean that the quality of care would be unequal and would depend on the training of the health professional the patient happened to encounter. Behind this idea, one can detect the feeling that the quality of care would vary with the social and economic capital of the patient, who would be more or less capable of having an informed opinion of the care situation he or she finds him or herself in. A certain level of social capital, which not all patients possess, would be needed to obtain access to those professionals considered the most competent when necessary.

It is in this sense that reference is made to the situation in Africa. Health professionals working in developing countries adapt their practices according to the professional staff available and to the means of accessing health care. Paradoxically, no mention was made in the interviews of examples drawn from models of practice in industrialized countries comparable with France, particularly in terms of health system organization (Bourgueil, Marek, & Mousquès, 2005), although these differences are known.

In reality, there are already situations (some reported by our interviewees) in which nurses evaluate a patient’s situation, develop a diagnostic hypothesis, form a judgment about the gravity of the signs and symptoms and about the urgency of intervention, and then decide whether to refer the case to a doctor. Their decision is more linked to questions such as the time available, remuneration, risk taking in terms of the regulations, and support of their medical staff colleagues, than to their actual capacities to evaluate the clinical situation being presented. The risks of lost opportunity for the patient, or of mistakes in diagnosis, were noted. These cases were put forward as potential risks in treatment by nurses, although there was also recognition that doctors can equally make mistakes (Fox, 1959).

### Aesthetic Aspects

The aesthetic aspect of the findings has to do with the mobilization of creative resources to transform an experience (Pépin, Kérouac, & Ducharme, 2010) and to offer new forms of meaning. Those interviewed identified some domains in which nurses have acquired particular skills, or where they could easily apply them. For example, nurses are recognized as having skills in performing “beautiful” dressings that doctors, as prescribers, are less good at. In addition, some interviewees suggested that carrying out certain activities or managing certain situations requires different levels of knowledge and that it is not always practical for doctors to do so.

The prospect of giving more autonomy to nurses by widening their sphere of competence and allowing them to make diagnoses and provide necessary care induces a confusion of roles for some interviewees. There is a feeling that such a measure would transform nurses into doctors, but this idea was rejected by both doctors and nurses. Greater autonomy is not necessarily interpreted as greater competence or as a validation of the experience and the content of the nurse’s vocation but as an encroachment on the territory of the doctor and as a change in profession. In contrast, some nurses referred to their quest for excellence in their own practice and to their love for their profession. They do not want to be converted into doctors, even if they are ready to upgrade their own practices to a more complex level of activity.

### Trust

For the purposes of our discussion, we must consider not only trust in decision makers and/or in institutions external to those directly involved in the practice of health care but also the trust between these health workers themselves. Relationships between professionals are couched in terms of collaboration, hierarchy, or even power, which are linked to their prerogatives.

Contextual obstacles are considered to be a lack of common understanding and of support from the hierarchy. The development of the nursing profession can only take place under certain conditions; there should be agreement between the different parties, trust and solidarity between professionals, appropriate training, or the creation of a new diploma qualification. There would also need to be a sufficient number of professionals in the health services to be able to carry out the reorganization of working practice and the sharing of activities between professionals in the best possible way.

### Knowledge

Wider recognition of current competencies would make the knowledge of nurses more visible, as well as the services they already deliver, including those for which they are not legally authorized and do not claim payment. Some of them would be capable of performing certain tasks but would refuse to do so because such tasks exceed their mandate. Enabling their skills to be applied in a better way would improve the effectiveness of the organization of health care. Some nurses do sometimes carry out activities that are supposed to be performed only by medical prescription, putting the interests of the patient first and supporting the interests of team spirit and cooperation between paramedical and medical professionals. These nurses then authorize the situation retroactively with the doctor. These are assumptions of responsibility that result in time saving and in improvements to the treatment patients receive. Such situations take the form of implicit recognition of the competence of nurses. When a doctor retroactively authorizes the action taken by a nurse, he or she does so on the basis of the nurse’s judgment, without examining the patient.

Some restrictions are difficult to understand and have nothing to do with knowledge. Nurses acting alone cannot administer any medicines, even those such as acetaminophen, which are freely available in pharmacies without prescription. Occasionally, under the cover of a hospital’s procedures, nurses are authorized to administer pain relief to a patient who is, for example, in a hospital waiting room. However, in health centers or private clinics, none of the nurses we met were permitted to do so.

Nevertheless, nurses are sometimes more competent than doctors on certain points, although this fact can remain unstated, as is shown by a study by Hughes (1988) in the United Kingdom:

Nurses use subtle non-verbal and cryptic verbal cues to communicate recommendations, which in retrospect appear to have been initiated by the doctor. This “game” ensures that open disagreement is avoided and carries advantages for both parties: the doctor gains from the nurse’s knowledge and experience, while the nurse gains increased self-esteem and professional satisfaction from her more demanding role. (pp. 1–2)

In the same way, research in Spain shows that nurses trained in the delivery of non-complex primary care have essentially the same rate of success in treating health problems as doctors (Begoña et al., 2013).

## Conclusion

A variety of arguments have been used for and against the move toward greater autonomy for nurses, and this movement, if it were to take place, would have to be done in such a way as to fulfill certain conditions. In overall terms, the arguments developed during our research correspond to demands relating to the transfer of skills and levels of training and to the exercise of one or another of the possible advanced forms of nursing practice (clinical specialist or “practitioner”), as well as to levels of pay and staffing. Provided that these requirements are met, health professionals would accept these changes. After discussion of the arguments in favor of advanced nursing practice and of the obstacles to front line care being carried out by nurses, the idea can be seen to be accepted, subject to certain conditions concerning training, respect for nursing prerogatives, and recognition of activities. There are different perspectives for further research. In France, this type of theme has not yet been explored, and there is a need for qualitative data to inform health policy makers. From an international point of view, practices in France are not well known, and this makes it difficult to make international comparisons. There is no clinical evaluation of the efficiency of nurses’ activities when performing advanced practice in France. It would be interesting to explore the patients’ points of view and the patient acceptability of the establishment of a consulting nurse role. The French health insurance system and the perspectives of health policy makers would be important to study. Research could be performed in individual organizations where nurses work alone.

Our study has some limitations. The fieldwork was wide in scope, covering different health organizations (emergency departments in hospitals, and private and public health structures). This variation hindered a more situated analysis, focusing on the situation of a particular organization.

In France, a common preconceived idea suggests that doctors and nurses are not ready to accept greater autonomy among nurses, extending their practice to front line care. This work has enabled us to overcome this obstacle and to explore possible future public health policies, new forms of professional organization, and new patterns of activities oriented to advanced practice, as well as the resistance that must be understood and considered. Although medical practice in France has been essentially individual, we can see a tendency for young doctors to opt increasingly for group forms of practice. We know that in countries where collective forms of medical organization are preferred, advanced practice is more established. This trend might have a positive impact on the acceptability of new forms of nursing practice.

## Appendix

### Guidelines for Nurses

What are your main clinical activities with patients in the service? Can you give some examples?
What are the key capabilities that you use to perform clinical activities with patients?
What led you to develop capabilities for your practice after obtaining the State diploma?
What are the capabilities that you think need to be developed to exercise your clinical activity?
Have you identified specific skills in some experienced nurses service? If yes, which?
Do you consider yourself to be particularly competent in a particular area? If so, which one?
If you had the opportunity to see patients and undertake their care without their being seen by the doctor, would you do it? If so, why and under what conditions? If not, why not?

### Guidelines for Doctors

What are the main activities performed by clinical nurses in the service?
What are the key capabilities used by nurses to perform their clinical work with patients?
What, in your opinion, is the capacity shortage for nurses to exercise their clinical activities?
Have you identified specific skills in some experienced nurses service? If yes, which?
If the option was given to skilled nurses, in a specific context, to see patients and undertake their care, without their being seen by a physician, do you agree that they should do so? If so, why and under what conditions? If not, why not?
